# What Is Heredity

**Published:** 1889-05

**Authors:** Richard Goodman


### WHAT IS HEREDITY.

It is the transmission of physical, mental and moral qualities from parents to offspring, and constancy in this transmission is the law summarized in the maxim that "like produces like," but the same experience which guarantees the constancy also teaches that this constancy is not absolute. Variations occur and this makes the problem of hereditary transmission, in itself, one of the most interesting of physiological problems, also one of the most baffling.

The doctrine of inheritance is an immense one and of unknown antiquity ; we see it in the historians' reference to the heritage of caste, and intellectual attributes and moral qualities among the most ancient nations. The poets and philosophers refer to it. Homer represents Minerva addressing Telemachus in language not inapplicable to the present age as respects its great men :

> "Few sons their fathers equal : most appear degenerate."

Owing, it is said to the unequal matches intellectual men make in marriage, as the influence of both parents centers in and molds the offspring ; and Horace in one of his odes says,

> "Ye Romans, ye though guiltless shall
> Dear expiation make for all
> The laws your sires have broke."

The Scriptures abound with the recognitions of moral heritage, and the second commandment enunciated the law of hereditary transmission as part of God's moral government, just as the Sabbath which was at the end of creation sanctified and has been observed by the Jews in their desert wanderings, is endorsed as a holy and permanent institution and as the bow which He had set in the cloud after the flood, was to become a sign of a solemn compact made with man by the Deity.

The lesson taught practically which the law afterward expressly enunciates that God visiteth the sins of the fathers upon the children, occurred early in the world's history in the case of Noah and his son Ham and grandson Canaan. And perhaps one of the most striking examples of hereditary transmission of race qualities appears in the history of the descendants of Jacob and Esau as pointed out by Dean Stanley in his history of the Jewish church. "We have," says the Dean, "in Esau the chieftain of Edom, the likeness of the fickle, uncertain Edomite, now allied, now hostile to the seed of promise,—the wavering unstable dynasty which came forth from Idumea. Herod the magnificent and the cruel, Herod Antipas who heard 'John gladly' and slew him, Herod

Agrippa, almost a Christian, half Jew and half heathen. A turbulent and unruly race so described by Josephus in his day, always hovering on the verge of revolutions, always rejoicing in changes, roused to arms by the slightest motives, rushing to battle as if they were going to a feast. On the other hand we cannot mistake the type of the Israelites in whom beyond even Abraham and Isaac they recognized the father of Israel. Jacob seems to have displayed the meaner characteristics which have given opprobrium to the whole race in modern times, and to have learned like maltreated animals to have the fear of man habitually before his eyes."

In Jacob we see the same timid, cautious, watchfulness that we know so well, though under darker colors, through our great masters of fiction in Shylock of Venice and Isaac of York. But no less in the noble side of his career, do we trace the unbroken endurance, the undying resolution which keeps the nation alive still, even in its present overcast condition and which was the basis in its brightest days of the heroic zeal, long suffering and hope of Moses, of David, of Jeremiah, of the Maccabees, of the twelve Jewish apostles and the first martyr Stephen, and it might be added of the ever enduring, ever suffering apostle to the Gentiles. This is not the place for attempting to elucidate the ethical reason for God's "loving Jacob and hating Esau," but the result shows that the "blessing" which God designed for Jacob in the beginning and which Isaac was determined to bestow on Esau, was providentially bestowed as intended, though by intervening agencies not to be commended—not the only instance of the secret purpose of the Creator being accomplished by methods, the reason for employing which, are to us inscrutable.

We now return to the earlier instance of Noah and his grandson Canaan as a peculiar case of hereditary transmission of evil qualities foreseen by the patriarch, who made the occasion of his son Ham's conduct a reason for disclosing the consequences to the remote generations of those evil tendencies inherited by his youngest son and in and through him enlarged and disseminated to his posterity.—RICHARD GOODMAN, *In Laws of Life.*

